# Empyema of the Antrum

**Published:** 1903-08

**Authors:** O. N. Heise

**Affiliations:** Cincinnati, Ohio


### EMPYEMA OF THE ANTRUM.[1]

BY O. N. HEISE, M.D., D.D.S., CINCINNATI, OHIO.

In an article on Empyema of the Antrum appearing in one of our leading dental journals not long ago I noticed a statement as follows: "Within the last few years a comparatively new affection, frequently confounded with antral empyema, has appeared; dentigerous cysts arise, as you know, about the tooth-roots, and slowly dilate the tissue at the root of the tooth." I quote this principally to show that affections of the teeth and alveolar process in relation to antral troubles do not receive the attention they should and deserve. If the author of the above referred to article had taken the trouble to inform himself, he would have found that it was not a new affection; also that a dentigerous cyst is a different condition of affairs than he states it to be.

These maxillary cysts, when found in the neighborhood of the molars and bicuspids, can and do in their development raise or push the floor of the antrum to such an extent that finally the thin intervening lamella of bone is resorbed, leaving only the periosteum and the lining mucous membrane of the antrum as a covering. Oftentimes I have seen the encroachment such as to include the entire antrum, filling it completely, in its further development raising both outer and inner wall as well as the roof.

Antral cysts developing from within the cavity must of necessity first completely fill it before they can produce any abnormality in the way of bulging of either wall, and can, I think, be recognized as having their origin from within, instead of having developed with the alveolar process. The facial wall being extremely thin, owing to the resorption of the bone, leaves at times a decided defect in the wall, whereas in the dental or alveolar origin the thinness of the wall is found to be in the alveolar region or process. They are more frequent than has been supposed, not only in connection with antral lesions, but in other regions of the alveolar process, both in the upper and lower maxilla. Many an abscess has been treated with little or no success, the tooth finally extracted, the socket curetted, packed, etc., thinking it to be a case

[1] Abstract of a paper read before the American Medical Association, Section on Stomatology, New Orleans, May 5 to 8, 1903.

of caries; whereas, if the proper diagnosis had been made, the tooth could easily have been saved by proper treatment, especially the single-rooted teeth.

The domain of the stomatologist runs very closely into that of the rhinologist. Every now and then in the treatment of an obstinate antral suppuration we find that the trouble is not so much in the antrum as in some of the accessory cavities draining into the antrum, making a reservoir, so to speak, of it, and were treating only the effect, not the true cause in such instances. This fact, and also the frequent occurrence of empyema after la grippe, has led some men to almost doubt the dental origin of antral empyema. There is no question at all in my mind that many antral cases are primarily due to infection from some accessory cavity, or other nasal disease; nevertheless dental causes are and will remain prominent etiological factors.

The difference of opinion as to the main causation of antral empyema depends greatly upon the view-point taken, reminding one of the Oriental story of the three blind men who went one day to inspect an elephant. "He is like a spear," said one, who grasped the tusk. "He is like a fan," said another, who felt his ear; while the third, with his hand on his leg, declared he was "like a tree." They had all inspected the elephant, but naturally formed different ideas of his appearance. So it is with the etiology of antral empyema, various observers form various views from various experiences, while if all would inspect each and every case, and judge of its cause and treatment on its own individuality, it would be seen that there is ample room for all opinions in considering this interesting subject.

It is not my intention to enter into a discussion as to which etiological factor, nasal or dental, is most frequently found to be the cause of antral suppuration, but it has seemed to me that of late the dental relationship to antral empyema has been somewhat overlooked, not only being frequently the cause, but having a decided influence in keeping a certain amount of irritation, thereby preventing a complete cure. I want therefore to bring this phase of the subject to your notice.

That many affections of the nasal cavity and antrum are due to diseased teeth, etc., is not to be doubted. Not only abscessed teeth, but oftentimes carious, and even filled teeth, especially the superior incisors, are the cause of nasal disturbances. Ziem, of

Danzig, was one of the first to point out the important relationship existing between diseased teeth and abnormalities of the nasal mucous membrane and antral troubles. He reports a number of cases showing that any carious tooth in the superior maxilla, and sometimes even in the inferior maxilla, can and does, by reflex action, bring about hypertrophica rhinitis, and sounds a note of warning against the imperfect treatment of carious and diseased teeth, especially the molars and bicuspids. It was due to his investigations that in the year of 1886 the real impetus to the study of antral empyema was given, showing conclusively that they were far more common than heretofore supposed or believed, Ziem himself being a sufferer from an antral empyema due to a diseased tooth. He also was the first to call or designate them as empyema of the antrum, which, however is not altogether a correct term, inasmuch as an empyema from a pathological stand-point is a collection of pus in an enclosed cavity of the body. In the so-called empyema of the antrum we have the collection of pus, but not in a completely enclosed cavity, as we have a continual or an occasional discharge of pus from it. It is useless, however, to find fault with the term until we have a better and more appropriate one to apply to it. Since Ziem's first important contribution to this subject and the other accessory sinuses of the nose, Zuckerkandl's numerous dissections, as well as P. Heymann, Moritz, Wolf, Harke, and others, it has been shown that diseases of the accessory sinuses are anything but infrequent; in fact, they are the rule almost, in acute infectious diseases, but fortunately will, if the natural openings are normal and not closed by some diseased condition of the nose, end in a spontaneous cure. That some do not end this way is due, according to M. Schmidt and E. Frankel, "to the varying virulence of the bacteria and the individual disposition of the patient."

It is, however, "rather strange," as pointed out by M. Schmidt, "that the various sinuses, being so frequently affected in cases of la grippe, should show the presence of the influenza bacillus so seldom,—only in one case out of thirty, according to E. Frankel's examinations."

Grunwald has well said, "The great frequency of inflammation of the antrum of Highmore, which follows from the anatomic position of the cavities, rendering them liable to infection by the extension of a process from the inferior turbinate, and favoring the isolation of the morbid process within them by the swelling of the surrounding tissues, and by the high position of the office above the floor of the cavity, is enhanced by the proximity of organs like the teeth that are so prone to become diseased. Accordingly, infection of the antrum of Highmore, derived from a diseased pulp by way of the lymph-channels, and catarrhal condition due to irritation accompanying coronal caries, are quite common, and, inversely, catarrh of the antrum may give rise to periodontitis and thus establish a vicious circle, so that the antrum disease persists even after other causes have been removed. As infection creeps along the lymphatics of healthy bone, a focus of infection in the crown of a tooth is by no means to be despised, for even if an empyema of the antrum be due to another cause, yet disease of the crown of a tooth is calculated to maintain such a state of irritation in the mucous membrane as may frustrate all attempts at a cure."

In an excellent article on the Heilbarkeit der Kieferhöhlenentzündungen, published in the *Archiv für Laryngologie und Rhinologie* (1889), Heft 3, Grunwald gives interesting and convincing statistics regarding the influence of the teeth in the treatment of antral affections,—namely, " In thirty-one antra of twenty patients who had sound teeth, or apparently so at least, thirty-nine per cent. were cured, twenty-three per cent. almost cured, sixteen per cent. improved, and twenty-three per cent. unimproved, whereas in twenty-eight antra of nineteen patients, in whom the decayed teeth influencing the condition of the antrum were extracted, sixty-five per cent. were cured, seventeen per cent. almost so, fourteen per cent. improved, and only five per cent. not cured."

Grunwald explains that in cases of those patients having apparently sound teeth there must have been some diseased condition present, in the way of a periodontitis, which was and is often overlooked in cases, even by dentists, as he on a number of occasions referred the patient to them, and they refused to extract the tooth or teeth on account of the apparent soundness of them; that the chances of overlooking any connection or influence that apparently sound teeth have in keeping up the suppurative process are much greater; it is easy to overlook a diseased condition of the roots of the teeth, whereas in the cases where teeth were found to be diseased and either acted as a direct cause of the empyema or were to a certain extent responsible for the original trouble in the antrum, or in keeping up the suppurative process, the extraction of them alone was a prime factor in the cure of the affection, but does not of itself, in the majority of cases, suffice to bring about a complete cure, inasmuch as the mere puncturing of the antrum does not show any brilliant results.

That not enough attention has been bestowed upon this matter is or has been shown by the fact that in a number of cases treated according to the accepted methods of to-day suppuration would persist until the teeth involved in keeping up the process had been extracted, showing conclusively that although they might not have been the original cause of the suppuration, nevertheless they were active factors in preventing a complete cure. He also cites a number of cases to show the important relationship existing between diseased teeth and suppuration of the antrum.

There seems, however, to be a decided difference of opinion between the anatomists and clinicians regarding empyema of dental origin, the former claiming, as no microscopic evidence of any inflammation or change of structure is found upon section of the antrum between the roots of the teeth and the cavity, that the teeth decayed or diseased play little if any active part in its production.

They do admit occasionally to have found sufficient dental cause. Zuckerkandl, in three hundred cases, could trace only one as being due to dental causes. The same with E. Frankel, while Dmochowsky never found any evidence. Hajek, in this connection, remarks, "This marked difference of opinion of anatomical and clinical evidence is due to the fact that in the course of time all evidence which would point to a disease of the alveolar process or teeth is or has been entirely lost, as it is quite possible for the germs to have penetrated through the alveoli of the teeth and the intervening bony plate, as is the case in an affection of the meninges in frontal sinus and sphenoidal empyema without leaving or producing any macroscopic changes in the bone."

Kyle states, "Too much importance cannot be attached to the teeth as a causal factor in antral lesions." A majority of cases he believes are due to diseases of the teeth, and gives the percentage as high as seventy; he also states that it may be a post-operative complication of nasal and dental surgery. One case observed by him was due to the use of arsenic applied for the destruction of a pulp in a decayed tooth. The application was made twice in three days, and not seen for several days afterwards, when the antrum was involved, and extensive tissue necrosis had occurred with infection (evidently due to a very careless application of the arsenic). In another case extensive necrosis and suppuration had followed the injection of chloride of zinc into a tooth-cavity which connected with the antrum.

The relationship between diseased conditions of teeth with the upper jaw, gums, alveolar process, and antral troubles, as well as some lesions of the nose, is an intimate one, and should be taken into consideration more frequently than it is, and, as Kyle has well said, "A thin alveolar process of upper jaw from lesions of the teeth may cause by extension of inflammation, by continuity of structure, lesions of the floor of the nose or of the antrum; on the other hand, deflections of the septum or spurs situated close to the floor of the nose, by the inflammatory action set up in the surrounding structure may bring about inflammation and diseased conditions of the teeth in the direct line of obstruction.

The stomatologist should therefore not only have a thorough knowledge of the nasal cavities and accessory sinuses, but also of general medicine, and, indeed, the general practitioner or specialist should have a more thorough knowledge of stomatology.

